# Exploring the effects of a family admissions program for adolescents with Anorexia Nervosa

**DOI:** 10.1186/2050-2974-3-S1-O48

**Published:** 2015-11-23

**Authors:** Keren Fink, Paul Rhodes, Stephen Touyz, Andrew Wallis, Jane Miskovic, Sloane Madden, Michael Kohn

**Affiliations:** 1 University of Sydney, Sydney, Australia; 2 Children's Hospital at Westmead, Sydney, Australia

## 

The present study aimed to qualitatively investigate patient experience and treatment efficacy of the Family Admissions Program (FAP) – a pilot treatment program being run at the Children's Hospital at Westmead for adolescents with Anorexia Nervosa (AN). Based on Maudsley Family Based Treatment (MFBT), the FAP involves an adolescent and his/her family undergoing a two-week family-based hospital admission at the outset of treatment. The program aims to increase intensity and support to a level needed by families at risk of responding negatively to traditional MFBT.

Narrative Inquiry and Interpretative Phenomenological Analysis (IPA) were used to analyse data obtained from participant interviews, exploring both the prospective expectations and retrospective experiences of families and clinicians participating in the program. Results from both of the qualitative analyses indicate that in cases where the family unit has been particularly fractured as a result of AN, the FAP offers an opportunity for relational strengthening and reunification, particularly within the parental subsystem. Combined with the program's intensive support, contained transition from hospital to home, and proximity to necessary hospital services, this serves to provide struggling families and parents with a more skilled and consolidated foundation for traditional outpatient MFBT.

